# The thalamic low-threshold Ca^2+^ potential: a key determinant of the local and global dynamics of the slow (<1 Hz) sleep oscillation in thalamocortical networks

**DOI:** 10.1098/rsta.2011.0126

**Published:** 2011-10-13

**Authors:** Vincenzo Crunelli, Adam C. Errington, Stuart W. Hughes, Tibor I. Tóth

**Affiliations:** Neuroscience Division, School of Biosciences, Cardiff University, Museum Avenue, Cardiff, UK

**Keywords:** thalamic neurons, cortical neurons, probabilistic network model, dendrites, intrinsic calcium signalling

## Abstract

During non-rapid eye movement sleep and certain types of anaesthesia, neurons in the neocortex and thalamus exhibit a distinctive slow (<1 Hz) oscillation that consists of alternating UP and DOWN membrane potential states and which correlates with a pronounced slow (<1 Hz) rhythm in the electroencephalogram. While several studies have claimed that the slow oscillation is generated exclusively in neocortical networks and then transmitted to other brain areas, substantial evidence exists to suggest that the full expression of the slow oscillation in an intact thalamocortical (TC) network requires the balanced interaction of oscillator systems in both the neocortex and thalamus. Within such a scenario, we have previously argued that the powerful low-threshold Ca^2+^ potential (LTCP)-mediated burst of action potentials that initiates the UP states in individual TC neurons may be a vital signal for instigating UP states in related cortical areas. To investigate these issues we constructed a computational model of the TC network which encompasses the important known aspects of the slow oscillation that have been garnered from earlier *in vivo* and *in vitro* experiments. Using this model we confirm that the overall expression of the slow oscillation is intricately reliant on intact connections between the thalamus and the cortex. In particular, we demonstrate that UP state-related LTCP-mediated bursts in TC neurons are proficient in triggering synchronous UP states in cortical networks, thereby bringing about a synchronous slow oscillation in the whole network. The importance of LTCP-mediated action potential bursts in the slow oscillation is also underlined by the observation that their associated dendritic Ca^2+^ signals are the only ones that inform corticothalamic synapses of the TC neuron output, since they, but not those elicited by tonic action potential firing, reach the distal dendritic sites where these synapses are located.

## 1. Introduction

**Figure 1.**
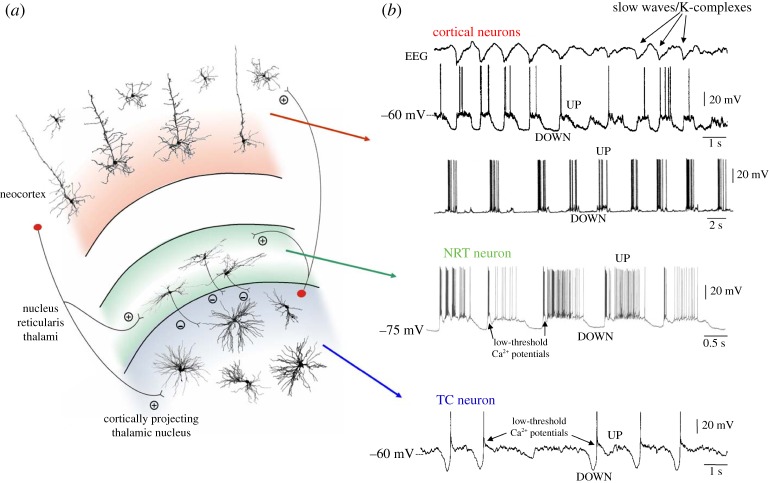
The EEG slow (<1 Hz) rhythm and its cellular counterpart, the slow (<1 Hz) sleep oscillation, in cortical and thalamic neurons. (*a*) Diagram of a TC module with its most relevant neurons and synaptic connections (thalamic interneurons and neocortical neurons other than those in layers 4 and 5/6 have been omitted for clarity). Plus and minus indicate excitatory and inhibitory synapses, respectively. (*b*) The slow (<1 Hz) rhythm in the EEG (top trace) and its cellular counterpart, the slow (<1 Hz) oscillation, recorded in two different cortical neurons and in a TC and NRT neuron *in vivo*. Black arrows in the NRT and TC traces highlight the low-threshold Ca^2+^ potential (LTCP) that is invariably present at the start of each UP state in thalamic neurons. Adapted with permission from Crunelli & Hughes [2].

The slow (<1 Hz) sleep rhythm is the most fundamental activity of non-rapid eye movement (NREM) sleep [1–4]. Its cellular counterpart, the slow (<1 Hz) sleep oscillation, occurs quasi-synchronously in all types of cortical neurons, in the glutamatergic thalamocortical (TC) neurons of various sensory and intralaminar thalamic nuclei and in the GABAergic cells of the nucleus reticularis thalami (NRT), and consists of regularly repeating depolarizing sequences (most often with firing) interspersed with hyperpolarizations (with no firing) at a low (0.2–0.9 Hz) frequency [5–7], nowadays commonly referred to as UP and DOWN states, respectively (figure 1).

The physiological importance of the slow (<1 Hz) oscillation is highlighted by its presence throughout all stages of NREM sleep both in humans [1,3,4,8,9] and in animals [10–13]. In fact, the electroencephalogram (EEG) K-complex (of stage 2 sleep) represents a single cycle of the slow oscillation, with its surface EEG-negative and -positive phases (corresponding to depth EEG-positive and -negative) simply reflecting the DOWN state and the start of the UP state, respectively, of the oscillation [14–16]. Sleep spindles (of stage 2–3 sleep) frequently occur immediately after, or are superimposed on, the depth-negative peak of the EEG wave, i.e. at the start the slow oscillation UP state [17–19]. As sleep deepens, the frequency of the slow oscillation, and thus that of K-complexes, increases until it develops into the slow waves of deep NREM sleep [19]. From stage 2 to 4 of natural sleep, therefore, the slow oscillation shows an increase in frequency (from around 0.03 to almost 1 Hz) and occupies an increasingly larger component of the EEG signal with clear periods of delta waves becoming more frequent and longer [14]. A slow oscillation with similar properties to the one observed during natural sleep is also observed during anaesthesia in various cortical and thalamic neurons [5,10,11,20–22] and importantly the characteristic increase in frequency that is present during the progression from light to deep natural sleep also occurs during the deepening of anaesthesia both in humans and in animals [14,23] (see [2]).

## 2. Mechanism of the slow oscillation

**Figure 2.**
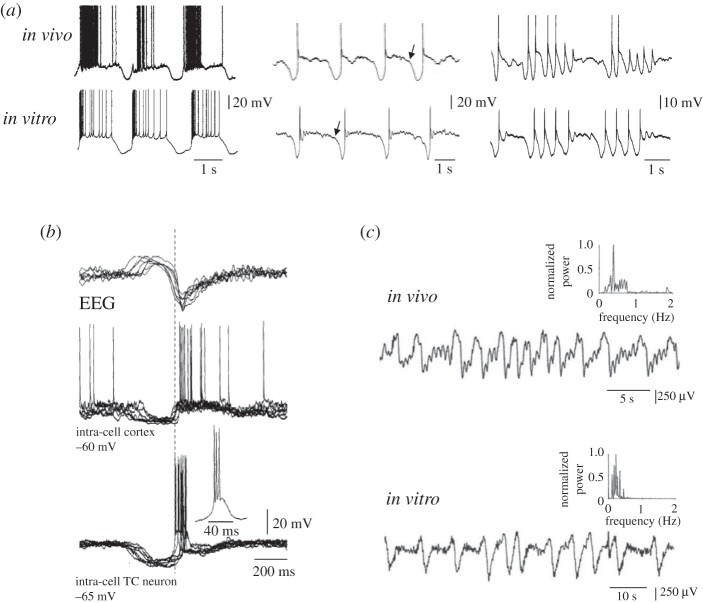
Similarity of the slow oscillation *in vitro* and *in vivo* and leading role of thalamic firing in the generation of a new UP state. (*a*) Examples of the slow oscillation recorded *in vitro* and *in vivo* in TC neurons. Note the strong similarities in the waveform of the slow oscillation recorded in the two experimental conditions. Traces *in vitro* were obtained in the presence of 100 μM *t*-ACPD. (*b*) Simultaneous EEG and intracellular recordings from a cortical and a TC neuron *in vivo* show the LTCP-mediated bursts of action potentials in the TC neurons to precede the UP state of the cortical cell. (*c*) Local field potentials recorded from the lateral geniculate nucleus *in vivo* during natural deep NREM sleep (top) and in a thalamic slice in the presence of *t*-ACPD (50 μM). Both traces show a characteristic slow rhythm at <1 Hz. The presence of this rhythm in a thalamic slice indicates that mechanisms exist within the thalamus to elicit a synchronized (<1 Hz) output even in the absence of a cortical input. Adapted with permission from Crunelli & Hughes [2].

Almost all neocortical neurons participate in the slow oscillation [5–7] (figure 1*b*), with their UP and DOWN states occurring in a quasi-synchronous manner among different types of cortical neurons, even between relatively distant cortical territories [13]. Interestingly, different types of cortical neurons appear to contribute to the network slow oscillation with their own particular signature, both in terms of action potential timing and with regard to their UP and DOWN state waveform [24,25]. *In vitro* studies [26–28], sophisticated analysis of *in vivo* data [29] and computer simulations [30–32] have demonstrated that the slow oscillation in neocortex mainly results from the regular recurrence of intense, but balanced, intracortical synaptic barrages, which generate the UP state, and their absence, which constitutes the DOWN state (figure 2*a*). However, although neocortical UP states are predominantly synaptically based, they are potentially aided by intrinsically oscillating (i.e. pacemaker) neurons, including a subset of pyramidal neurons in layers 2/3 and 5, and a group of Martinotti cells in layer 5 [33].

As with neocortical cells, almost all TC neurons, both in sensory (figure 2*a*) and intralaminar nuclei, as well as NRT cells exhibit the slow oscillation with their UP and DOWN states occurring quasi-synchronously, both locally and with cortical neurons [2,5,34,35]. In contrast with neocortical neurons, however, the slow oscillation recorded in both TC and NRT neurons either *in vivo* or *in vitro* shows a stereotypical waveform from cycle to cycle (figure 2*a*) [34–38]. In particular, there is a large (15–20 mV) and constant voltage difference between the UP and DOWN states, and the evolution of the membrane potential during the slow oscillation is characterized by two distinctive signatures at the transition between states. Specifically, the transition from DOWN to UP state is punctuated by a low threshold Ca^2+^ potential (LTCP) and associated high frequency (150–300 Hz) burst of action potentials (figure 2*a*), whereas the transition between the UP and DOWN state is marked by a clear inflection point in the membrane potential (arrows in figure 2*a*).

The presence of these peculiar similarities in the slow oscillation across thalamic cells and nuclei, its insensitivity to tetrodotoxin and to blockers of ionotropic GABA and glutamate receptors, the ability of thalamic slices to generate a large field potential (at <1 Hz) similar to those observed *in vivo* (figure 2*c*), and the strong dependence of the slow oscillation frequency on membrane polarization (see [2]) strongly argue for the slow oscillation in TC and NRT neurons as being a pacemaker activity. In fact, it results from the membrane potential bistability that is generated by the interplay of *I*_Leak_ and the window component of the low threshold Ca^2+^ current *I*_T_ (*I*_Twindow_) [36–39], such that the UP state essentially corresponds to the condition when *I*_Twindow_ is active and the DOWN state to when *I*_Twindow_ is inactive [40,41]. Other membrane currents that are essential for the expression of the slow oscillation in TC and NRT neurons include *I*_CAN_ (Ca^2+^-activated, non-selective cation current) and *I*_h_ (hyperpolarization-activated, Na^+^/K^+^ current) [2], which in the absence of synaptic inputs are the main determinants of the duration of the UP and DOWN states, respectively. In NRT neurons, the slow oscillation is also shaped by Ca^2+^- and Na^+^-activated K^+^ channels [36].

## 3. Temporal dynamics of the slow oscillation in cortical and thalamic neurons

The *in vitro* evidence, suggesting that isolated thalamic neurons [36,37] and networks [2,42] are capable of independently producing the slow oscillation, appears at first sight to be in contrast with the *in vivo* data showing that the slow oscillation is abolished in TC and NRT neurons following decortication or transection of the cortical afferents [5,43,44]. In these lesion-based studies, however, no systematic and quantitative comparison of the properties of the slow oscillation in the cortex before and after lesioning was undertaken. Moreover, a localized pharmacological block of intracortical connectivity *in vivo* has little effect on the long-range cortical coherence of the slow oscillation [43], whereas intrathalamic application of muscimol almost completely abolishes slow oscillations in individual rat cortical neurons *in vivo* [21]. Consistent with these *in vivo* findings, in TC slices where viable corticothalamic (CT) and TC connections are preserved, sectioning both afferents leads to a decreased occurrence of cortical UP states [45].

If the full manifestation of the slow oscillation requires the entire TC network, it then becomes important to understand the relative contribution of cortical and thalamic neuron firing to initiating a new UP state in a TC module. Although no study has directly addressed this issue, the original work on the slow oscillation clearly shows that the LTCP-mediated burst of action potentials that invariably marks the beginning of each TC neuron UP state *in vivo* can precede by 20–50 ms the start of the UP state in simultaneously recorded cortical neurons and the peak negativity of the depth-EEG wave [34] (figure 2*b*), suggesting that these bursts may be responsible for triggering UP states in the neocortex. The following lines of evidence also strongly support the idea that LTCP-mediated bursts of action potentials in TC neurons may act as a signal for initiating new UP states in the neocortex:

— thalamic volleys, even at frequencies lower than that of an LTCP-mediated burst, are the most effective way of eliciting cortical UP states *in vitro* and *in vivo* [46–50];
— spontaneous LTCP-mediated thalamic bursts powerfully activate neurons in the primary somatosensory cortex *in vivo* [51];
— whisker stimulation delivered during the DOWN state is highly effective in triggering UP states in layer 2/3 neurons *in vivo*[48];
— in cortico-thalamo-cortical slices, a brief train of stimuli that apparently produce an LTCP-mediated burst reliably triggers a cortical UP state whereas a single stimulus does not [49];
— also in cortico-thalamo-cortical slices, the TC neuron firing precedes the onset of cortical UP states in a large number of cases despite these measurements being biased against the thalamus because the start of the cortical UP state was defined as the onset of the local field potential and not at the cellular level [45];
— localized electrical stimulation close to the recording site or in layer 2/3 blocks existing UP states in layer 4/5 neurons*in vitro*, and is unable to evoke new ones except at low stimulation intensities [50];
— while cortical stimulation at low intensity enhances the ability of thalamic stimuli to evoke UP states, it reduces it at high intensity [45];
— whereas the slow oscillation recorded *in vitro* from TC neurons shows a characteristic increase in frequency (i.e. from 0.03 to 1 Hz) [2,36,37] that closely matches that observed during both the natural progression from light to deep NREM sleep and the deepening of anaesthesia [14], modelling studies of isolated cortical networks appear to show opposite results [30,31]; and
— the consistency of the K-complex and slow sleep rhythm EEG waveform across oscillation cycles during the progression of natural sleep and the deepening of anaesthesia matches more closely the uniformity of the corresponding DOWN to UP state transition and the duration of the DOWN state in TC than in cortical neurons [2].

**Figure 3.**
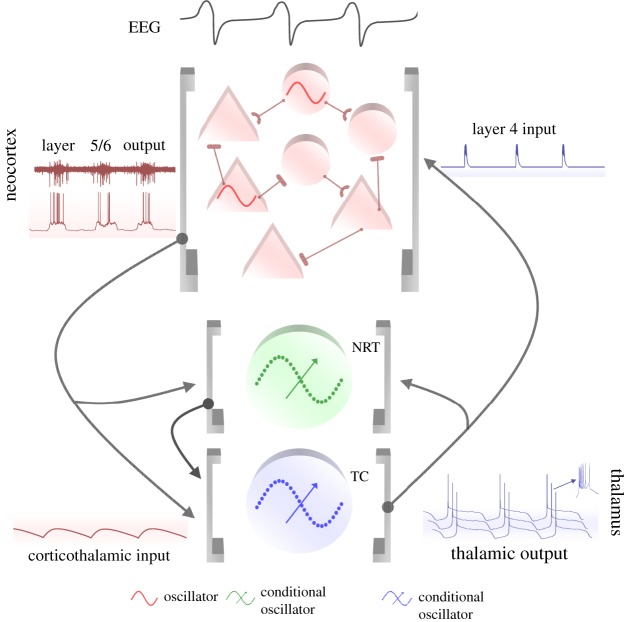
Schematic flow of the slow oscillation in a TC network. The prolonged UP states of the slow oscillation in layer 5/6 cortical neurons lead to long-lasting CT EPSPs (mediated by AMPARs, NMDARs and mGluRs in TC and NRT neurons). These slow EPSPs lead to an mGluR-induced reduction in *I*_Leak_ that is the necessary condition for thalamic neurons to exhibit the slow oscillation. The LTCP-mediated high-frequency burst that is invariably present at the start of each UP state of the TC neuron slow oscillation leads to highly effective bursts of TC EPSPs that initiate a new UP state in NRT and layer 4 neurons. The overall UP and DOWN state dynamics of a cortical region are maintained by synaptically generated barrages of excitation and inhibition from other cortical neurons but are also potentially fine tuned by additional intracortical inputs from intrinsically oscillating neurons in layer 2/3 and 5. Adapted with permission from Crunelli & Hughes [2].

In summary, therefore, although complex interactions among localized intracortical neuronal ensembles (e.g. the neocortical slice) allow the existence of the slow oscillation in cortical territories separated from the thalamus, thalamic inputs *in vitro* and *in vivo* appear as efficient and reliable a way of triggering UP and DOWN state dynamics in cortical networks as intracortical stimulation. On the basis of these findings and in view of the fact that both single TC and NRT neurons can produce the slow oscillation working as ‘conditional oscillators’ [2],^1^ we have recently proposed (i) that the full expression of the slow sleep rhythm in the EEG is generated by the balanced interplay of cortical and thalamic pacemakers (figure 3) and (ii) that the LTCP-mediated burst of action potentials that is invariably present at the start of each TC neuron UP state represents a major trigger for new UP states in the somatotopically linked cortical territory [2]. Indeed, preliminary *in vivo* evidence (supported by modelling studies) has conclusively shown that temporary inactivation of the thalamus results in the majority of cortical neurons being unable to exhibit the slow oscillation, while the others only express occasional non-synchronized UP states [55–57].

## 4. The slow oscillation in a thalamocortical network model

**Figure 4.**
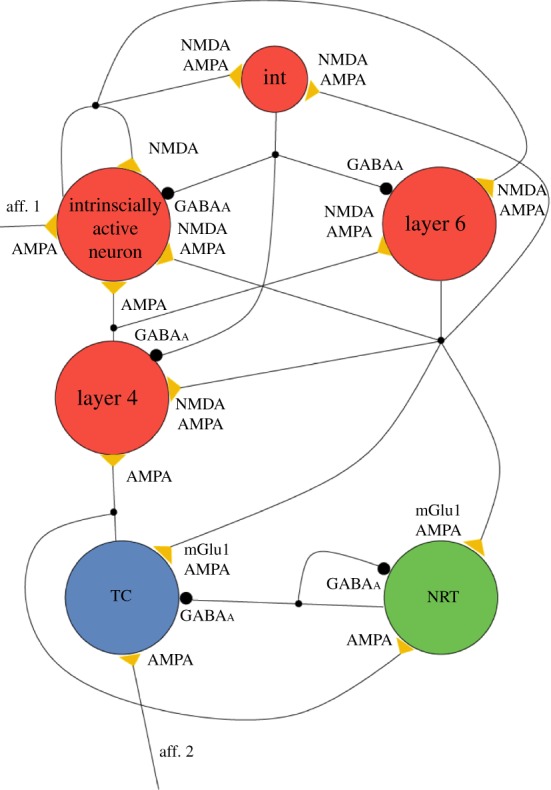
Topology of the TC network model. The model consists of six neuron pools as indicated (TC: thalamocortical neurons; NRT: neurons of the nucleus reticularis thalami; int: inhibitory interneurons; other neuronal pools as indicated). Yellow triangles and large black circles indicate excitatory and inhibitory synaptic connections, respectively, with their postsynaptic receptors. The small black circles on the connections represent branching points (i.e. origins of collaterals). Other crossings of the connection pathways do not indicate branching. (See appendix A for additional information.)

To test these hypotheses, we have used a TC network model (figure 4; see also appendix A) and investigated the activity of thalamic and cortical neurons during simulations of the slow oscillation. In view of the available evidence, our model is assembled so that the isolated cortical network (that includes interneurons, putative thalamo-recipient layer 4 and thalamo-projecting layer 6 neurons) is capable of eliciting the slow oscillation even in the absence of a thalamic input. Similarly, individual TC and NRT model neurons are constructed as ‘conditional oscillators’ with the slow oscillation being dependent on the *I*_Twindow_-based bistability mechanism that we have previously described for these neurons.

**Figure 5.**
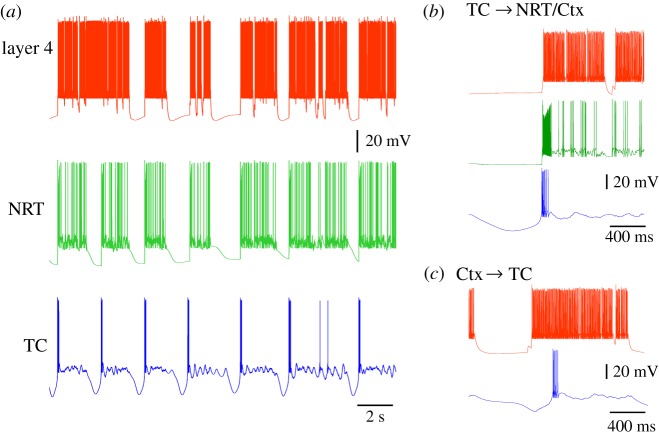
Simulated slow oscillation in the TC network model. (*a*) Simulated slow oscillation in layer 4, NRT and TC neurons. All UP and DOWN states occur simultaneously in the three cellular types except for the first DOWN state which is missing in the layer 4 neuron. (*b*) Traces of simulated slow oscillation showing the LTCP of the TC neuron to start the UP state in layer 4 and NRT neurons. Colour code as in (*a*). (*c*) Example of a layer 6 neuron UP state (red trace) preceding the UP state in a TC neuron (blue trace). This situation occurred much less frequently than that shown in (*b*).

The TC model satisfactorily reproduces the slow oscillation in individual cortical and thalamic neurons with characteristics similar to those observed *in vitro* and *in vivo*. In particular, quasi-synchronous UP and DOWN states in layer 4 and 6 cortical neurons and in TC and NRT neurons are commonly observed (typical frequency range: 0.25–0.35 Hz for different initial seed values; figure 5*a*). Importantly, the UP state of layer 4 neurons is triggered either by the LTCP burst of the TC neurons (figure 5*b*) or the firing of layer 6 cells (i.e. the thalamo-projecting neuron; figure 5*c*), though the former case is much more common (74–100%, in different simulations depending on the starting condition and the synaptic strength of the cortical afferents to TC and NRT neurons). The UP state in the NRT neurons is always triggered by the LTCP-mediated burst that occurs at the start of the TC neuron UP state. These results support our hypothesis that a quasi-synchronous slow oscillation can be generated by a TC network with thalamic neurons operating as ‘conditional oscillators’, and that the LTCP burst that is invariably present at the start of each UP state in TC neurons is by far the most frequent trigger of new cortical UP states.

### (a) Effects of blocking the corticothalamic input to thalamocortical neurons

In order to gain a deeper insight into the temporal dynamics of, and the network rules that control the slow oscillation in, TC networks, additional simulations were carried out where different synapses or their component postsynaptic receptors were individually blocked, starting first with the CT synapses on either TC or NRT neurons, then with the TC synapses on layer 4 cells.

**Figure 6.**
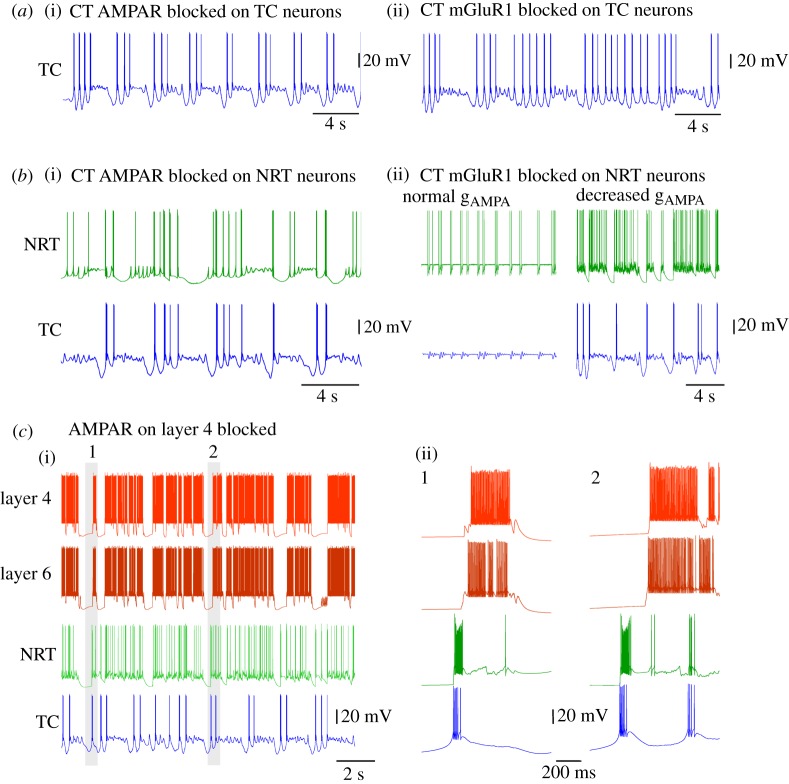
Effect of selective removal of postsynaptic receptors at cortical and thalamic synapses on the simulated slow oscillation. (*a*(i)) Simulated slow oscillation in a TC neuron with the CT AMPAR blocked shows groups of delta oscillation in the DOWN state. (ii) When the mGlu-R1 component of the CT synapses on TC neurons is blocked, the simulated slow oscillation in TC neuron is almost fully abolished and replaced by nearly continuous delta oscillation. (*b*(i)) Reduction in the number of synchronous UP and DOWN state transitions between NRT and TC neurons is observed when the CT AMPARs on NRT neurons are blocked. Also, the firing of the NRT neurons becomes much less intense (cf. figure 5*a*). (ii) Removal of the mGluR1 component of the CT synapse of NRT neurons brings both TC and NRT neurons into a permanent UP state with synchronized subthreshold oscillation and intermittent firing, respectively (normal g_AMPA_). A reduction in g_AMPA_ of the CT synapses on both TC and NRT neurons restores the slow oscillation (decreased g_AMPA_). (*c*) Block of the AMPARs on the thalamic afferent to layer 4 neurons leads to an almost complete loss of global synchrony across the TC network. At the same time, two separate synchronies emerge: one between layer 4 and layer 6 neurons and another between TC and NRT neurons. These can occur almost simultaneously (i) or clearly separated in time (ii).

When the AMPA receptor (AMPAR) component of the CT synapses on TC neurons is blocked (leaving both the mGluR component to TC neurons and the CT synapses on NRT cells intact), the degree of synchrony in the slow oscillation between TC, NRT and layer 4 neurons markedly decreases such that the number of UP states occurring synchronously are reduced to 40% of the control condition. Interestingly, under these conditions the waveform of the slow oscillation remains qualitatively similar in all cell types in the network, except in TC neurons where, in a substantial number of slow oscillation cycles, the DOWN states can exhibit groups of approximately 1–2 Hz delta oscillations (figure 6*a*(i)). This manifestation of the slow oscillation in TC neurons can also occur *in vivo* and *in vitro* (right traces in figure 2*a*) and studies in thalamic slices have additionally shown it to be due to a slight reduction in the steady depolarizing drive required to exhibit a ‘pure’ slow oscillation (see fig. 3*b* in [2]). Thus, our simulation results in a TC network model are consistent with *in vivo* and *in vitro* observations and show that solely removing the CT AMPAR drive to TC neurons significantly alters the nature of the slow oscillation DOWN state in these neurons without abolishing the oscillation itself.

When the mGluR component of the CT synapses on TC neurons is blocked (leaving the AMPAR component intact), the slow oscillation in TC neurons is almost fully abolished and replaced by nearly continuous delta oscillation (figure 6*a*(ii)), as expected from the essential contribution of this glutamate receptor subtype to the generation of the slow oscillation in TC neurons. Finally, when both AMPARs and mGluRs are blocked, only continuous delta oscillations are observed in TC neurons while the slow oscillation continues in cortical neurons, a result that is consistent with the *in vivo* studies showing an identical effect when the thalamus is disconnected from the cortex [43,44].

Interestingly, the activity of NRT neurons remains almost unaffected by changes in the CT synapses on TC neurons, except when both AMPARs and mGluRs are blocked: in this case NRT neurons entered into a permanent UP state with some occasional firing (not shown). This shows that abolishing the slow oscillation in TC neurons brings a similar result in NRT neurons albeit through a tonic depolarization that is brought about by an enhanced excitation of these cells that probably originates from the continuous rhythmic bursting of TC neurons.

### (b) Effects of blocking the corticothalamic input to nucleus reticularis thalami neurons

Next, the cortical input to NRT neurons was blocked, while leaving that on TC neurons intact. When only the AMPARs are blocked, a quasi-synchronous slow oscillation continues to exist in TC, NRT and layer 4 neurons. However, there is a clear reduction in synchrony between the two thalamic cell types such that the DOWN states are often no longer synchronized and the firing in the UP states of the NRT neurons is diminished due to the lack of AMPAR-mediated excitatory drive (figure 6*b*(i)). When the mGluRs on the NRT neurons are blocked (leaving the AMPARs intact), both TC and NRT neurons enter into a permanent UP state in which occasional small-amplitude subthreshold oscillations (in TC neurons) (figure 6*b*) and intermittent firing (in NRT neurons) occur, often in a synchronized manner between the two cell types (figure 6*b*(ii), normal g_AMPA_). Because a subsequent decrease in the strength of the CT afferents to both TC and NRT neurons rescues the slow oscillation in both cell types (figure 6*b*(ii), decreased g_AMPA_), it would appear that the occurrence of the continuous depolarized state in these cells can be explained by a relative increase in the AMPAR-mediated excitation that impinges on these cells following the block of mGluRs on NRT neurons. Such an increase results because of a complex reconfiguration of cortical network activity that accompanies this block: essentially a reduced activity of NRT neurons resulting from an initially *smaller* net excitatory drive from the cortex leads to a disinhibition of TC neurons which in turn leads *eventually* to an increased excitation of the neocortex and corresponding stronger AMPAR drive to both NRT and TC neurons. Finally, when both the AMPARs and mGluRs postsynaptic to the cortical afferents were simultaneously blocked in NRT neurons, TC and NRT neurons continue to exhibit slow oscillations, albeit with a reduced degree of synchrony and with the amount of firing present during the UP states in NRT neurons greatly diminished (as in figure 6*b*(i)).

### (c) Effect of blocking thalamic afferents to layer 4 cortical neurons

Having examined the effect of manipulating the cortical input to TC and NRT neurons on the expression of the slow oscillation we turned our attention to the role played by the AMPAR-mediated thalamic input to layer 4 cortical neurons. When this input is blocked, the triggering role of the LTCP-mediated bursts of TC neurons on the cortical slow oscillation almost fully disappears and the synchrony between cortical and thalamic territories ceases to exist (figure 6*c*). Instead, two separate synchronies emerge: one between TC and NRT neurons, in which the NRT neuron activity is directly triggered by the TC neuron firing, and the other between layer 4 and 6 neurons, with the start of firing in layer 6 cell UP states always preceding that in layer 4 cells. These two synchronies could either occur almost simultaneously (figure 6*c*(i)), or clearly separated in time (figure 6*c*(ii)). Thus, the activity of both layer 4 and 6 neurons continues to alternate between UP and DOWN states, similar to that in control conditions (cf. figure 5) but now independently of the activity of the thalamic network.

### (d) Summary of simulation results

Taken together, these simulation results validate both our original hypotheses [2]. Firstly, it is clear from simulations involving a block of CT inputs as well as when the TC input is removed that a full synchronous expression of the slow oscillation can only occur when the TC network is completely intact and therefore emerges from the balanced interaction between the cortex and thalamus. Secondly, the results of simulations whereby the thalamic input to layer 4 neurons is removed plainly show that the LTCP-mediated burst of action potentials that is present at the start of each TC neuron UP state represents the primary trigger for initiating new UP states in both the neighbouring inhibitory neurons of the NRT as well as neurons and networks in associated cortical territories. Lastly, the sometimes non-trivial effects of altering the nature of cortical input to thalamic neurons, such as the transition to a fully activated or continuous UP state in TC and NRT neurons that occurs following a block of mGluRs on NRT neurons, speak for the substantial complexity of network interactions and intricate interdependence of different elements that are at work in the TC network during the slow oscillation, a situation which closely replicates the one that is present in the real circuitry *in vivo*. As mentioned earlier, preliminary *in vivo* and modelling evidence indicates that in the absence of thalamic input the neocortex loses its ability to generate the slow oscillation [52–54].

## 5. Low-threshold Ca^2+^ potential invasion of thalamocortical neuron distal dendrites

**Figure 7.**
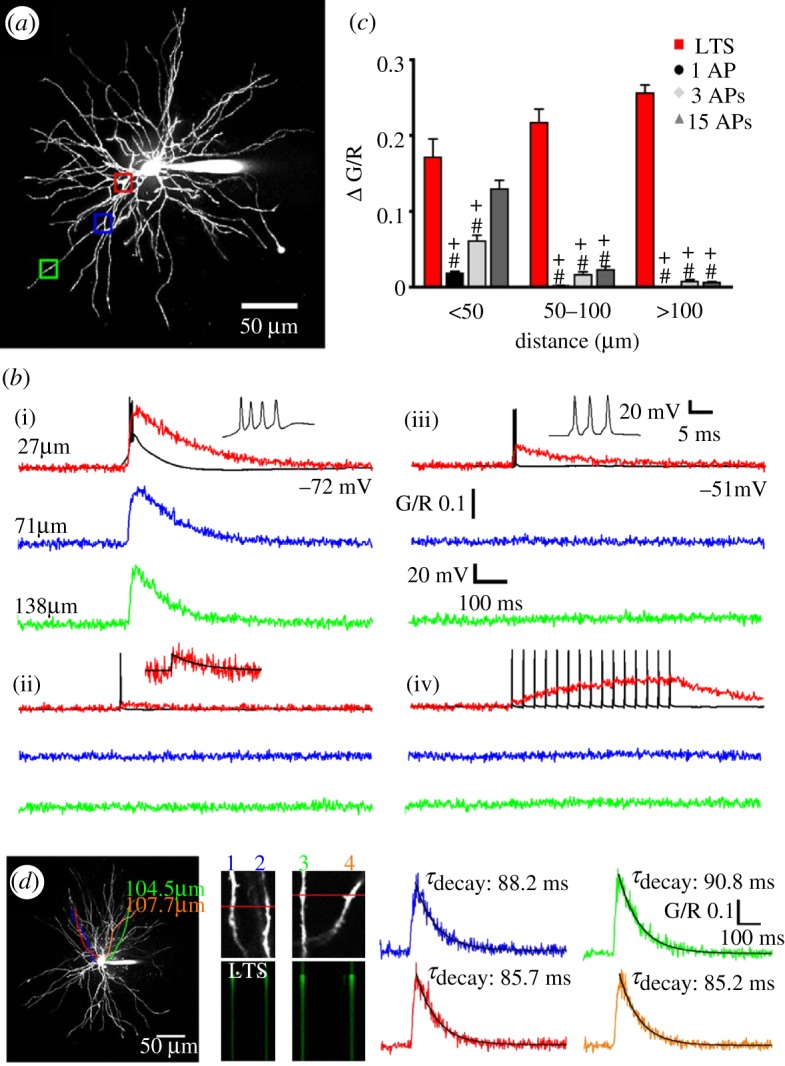
Intrinsic Ca^2+^ signalling in TC neuron dendrites. (*a*) Maximum intensity projection of a dorsal lateral geniculate TC neuron illustrating the dendritic sites where linescans shown in (*b*) were performed. Proximal, intermediate and distal dendritic locations are colour coded red, blue and green, respectively. (*b*) Dendritic Δ[Ca^2+^] evoked by (i) LTCPs, (ii) single backpropagating action potentials, (iii) three backpropagating action potentials at 200 Hz (mimicking the high frequency burst elicited by an LTCP) and (iv) 15 backpropagating action potentials at 30 Hz are shown overlaid onto somatically recorded voltage traces (in black). A single action potential-elicited Ca^2+^ transient is shown enlarged for clarity in (ii), with a mono-exponential fit shown in black. (*c*) Summary of Δ[Ca^2+^] amplitudes grouped by dendritic location. *p<0.05 (versus proximal Δ[Ca^2+^]LTCP), ^#^*p*<0.001 (versus proximal Δ[Ca^2+^]LTCP), ^+^*p*<0.001 (versus Δ[Ca^2+^]LTCP for each dendritic location) (*n*=6–11 cells). (*d*) Somatically evoked LTCPs produce synchronous Ca^2+^ transients throughout the entire dendritic tree. Two different pairs of distal dendrites (100–110 μm from the soma) lying in the same focal plane but originating from different stem dendrites (coloured tracings constructed from three-dimensional *Z* series) were imaged separately during evoked LTCP activity. In both pairs of dendrites, the LTCP-elicited Δ[Ca^2+^] occurred simultaneously in all four dendrites (amplitude and *τ* of the decay of the evoked Ca^2+^ transients are identical). Adapted with permission from Errington *et al*. [61].

The significance of the LTCP-mediated bursts of action potentials at the onset of the TC neuron UP states, however, may not be simply restricted to their ability to drive cortical UP states but could also carry additional significance for the computational dynamics of TC network activity during the NREM sleep stages where these burst most frequently occur [2]. In order to fully appreciate such potential roles it is important to stress two important features of TC neurons. Firstly, the glutamatergic synaptic inputs are differentially distributed across the TC neuron dendritic tree, with the sensory afferents arriving on stem dendrites close to the first dendritic branch point (less than 50 μm) and the more abundant CT fibres forming synapses mainly onto intermediate or distal dendrites (approx. 70–150 μm) [58–60]. Secondly, the changes in intracellular calcium ([Ca^2+^]) signalling that are elicited by action potentials and LTCPs in TC neurons show a high degree of dendritic region-specificity [61]. Thus, whereas the increase in [Ca^2+^] associated with single, or trains of, backpropagating action potentials across a wide range of physiological frequencies (1–300 Hz) is restricted to proximal sites (less than 80 μm), [Ca^2+^] signals elicited by an LTCP invade distal dendritic regions because of the widespread dendritic distribution of T-type Ca^2+^ channels in TC neurons (figure 7*a*–*c*) [61]. Indeed, with regard to either synaptically or somatically evoked LTCP, individual TC neurons essentially behave as global Ca^2+^ signalling units showing a near-instantaneous increase in [Ca^2+^] (figure 7*d*) [61].

Significantly, the fast extrusion of Ca^2+^ during LTCPs [61] together with the refractoriness of CaV3.1 T-type Ca^2+^ channels [62] would easily allow a non-summating global dendritic Ca^2+^ signal at the start of each UP state, and even following the short period of delta oscillation that at times is present during the DOWN state (cf. figure 2*a*). Another major consequence of these results is that whereas backpropagating action potentials can faithfully inform the proximal sensory but not the distal CT synapses of the TC firing output, CT synapses will only ‘detect’ [Ca^2+^] signals associated with LTCPs. In other words, the highly decrementing action potential backpropagation in proximal dendrites during TC neuron UP states will allow distal processing of CT inputs to proceed uninfluenced by this neuronal firing output except when an LTCP is generated, i.e. at the start of a new UP state. It is therefore probable that the LTCP which is invariably present at the start of a new TC neuron UP state may be responsible for creating both an electrical and a biochemical ‘window’ for plastic changes during a critical portion of the slow oscillation in these cells, as we have recently proposed [2].

## 6. Conclusion

The full expression of the slow (<1 Hz) sleep rhythm in the EEG requires the fine interplay of cortical and thalamic oscillators with the LTCP-mediated burst at the onset of the TC neuron UP states providing a robust signal for the start of a new UP state in somatotopically linked cortical territory and for a global increase in intracellular Ca^2+^ across the entire TC neuron dendritic tree.

## Footnotes

1 A slow oscillation with characteristics identical to those observed *in vivo* is observed *in vitro* under control conditions in a small number (5%) of TC and NRT neurons [52,53], and in almost all mouse, rat and cat TC and NRT neurons when the function of the metabotropic glutamate receptors (mGluRs) that are located postsynaptically to their cortical afferents (i.e. mGluR1a) [54] is re-instated either pharmacologically (by exogenous application of an mGluR1a agonist) or physiologically (by the glutamate released following electrical stimulation of the corticofugal afferents that are present in the thalamic slice) [36–38]. Thus, a cortical slow oscillation drive is not necessary for the expression of the slow oscillation in either TC or NRT neurons. Instead, the slow oscillation in thalamus can be generated by each TC and NRT neuron operating as a ‘conditional oscillator’, i.e. by the dynamic interplay of their voltage-dependent membrane currents (mainly *I*_Twindow_ and *I*_Leak_) [39], with sustained mGluR1a activation providing the necessary ‘condition’ for these oscillators to work [40,41].

## Appendix A

(a) The thalamocortical network model

Our TC network model is based on the notion of the ‘average neuron’ (fully described in [63]), since this type of model allows an easier implementation of detailed Hodgkin–Huxley-type neuron models in large networks [64]. Here, therefore, we only list the specific properties of the TC network model as used in this study. The TC network model consists of 6 neuronal pools (figure 4): TC neurons, NRT neurons, cortical layer 4 neurons, cortical layer 6 neurons, cortical inhibitory interneurons and intrinsically active neurons, similar to that described in Compte *et al*. [31]. To ensure the robustness of the observed results, for a given parameter set simulations were run using at least two different initial conditions.

The thalamic section of the model consists of a TC neuron pool (with 300 identical single-compartment models of TC neurons) and an NRT neuron pool (with 250 identical NRT single-compartment models). Their set of intrinsic and synaptic currents is illustrated in tables 1 and 2, respectively. In particular, each TC neuron model includes AMPAR- and mGluR-containing synapses from the layer 6 neuron pool and GABA_A_R-mediated synapses from the NRT neuron pool. Each NRT neuron model includes AMPAR- and mGluR-containing synapses from the layer 6 neuron pool and AMPAR-containing synapses from the TC neuron pool. Both TC and NRT models were tested and validated for their ability to satisfactorily reproduce the slow oscillation by comparison with available *in vitro* experimental data [36–38,56].

**Table 1.** Voltage-gated conductances in the different neuron models. All conductances are expressed in nS. gL, leakage current; gNa, fast inactivating Na^+^ current; gK, delayed rectifier K^+^ current; gT, T-type Ca^2+^ current; gCaL, L-type Ca^2+^ current; gH, hyperpolarization-activated Na^+^/K^+^ current; gKCa, Ca^2+^-activated and voltage-gated K^+^ current; gCAN, Ca^2+^-activated cation current; gKNa, Na^+^-activated K^+^ current. — indicates the lack of a conductance in the neuron model.

	gL	gNa	gK	gT	gCaL	gH	gKCa	gCAN	gKNa
layer 4	5	1800	997.1	—	62	32.5	2.6	—	—
intrinsically active neuron	5	1712.3	997.1	—	35	32.5	—	—	—
layer 6	5	1800	997.1	—	62	32.5	2.6	—	—
interneuron	17.7	1712.3	997.1	—	35	32.5	15	—	—
TC	3	2893.2	507.3	74.6	5.5	74.3	—	25.1	—
NRT	2.5	964	180	17	—	8.0	3	20	25

**Table 2.** Parameters of the synaptic models. The rows show the neuron pool from where the synapses originate, the columns where they converge, while symbols indicate the type of receptor at a given synapse (N: NMDA; A: AMPA; G: GABA_A_; mG: mGlu). Values in brackets represent the number of synapses and their unitary conductance, respectively. — indicates the lack of connection between two neuron pools. All conductances are expressed in nS, except for mGlu which is expressed as an amplification factor (see text for details).

	layer 4	intrinsically active neuron	layer 6	interneuron	TC	NRT
layer 4	—	A:(20,1.0)	N:(3,1.6) A:(2,2.5)	—	—	—
intrinsically active neuron	—	N:(3,1.4)	N:(14,1.6) A:(10,2.5)	N:(5,1.6) A:(20,2.5)	—	—
layer 6	N:(30,1.6) A:(10,2.5)	N:(3,1.6) A:(10,2.5)	—	N:(6,1.6) A:(8,2.5)	A:(7,0.2) mG:(20,5.0)	A:(10,0.24) mG:(25,5.0)
interneuron	G:(10,1.14)	G:(7,1.14)	G:(18,1.17)	—	—	—
TC	A:(20,1.4)	—	—	—	—	A:(10,1.4)
NRT	—	—	—	—	G:(10,0.4)	G:(19,1.17)
aff. 1	—	A:(20,2.5)	—	—	—	—
aff. 2	—	—	—	—	A:(20,2.5)	—

Each of the four cortical neuron pools has 400 neurons, and contains a different array of intrinsic and synaptic currents which are listed in tables 1 and 2, respectively. In particular, the cortical interneuron model makes GABA_A_R-mediated synapses with all other three types of cortical neuron pools and receives AMPAR and NMDAR synapses from the cortical layer 6 and the intrinsically active cortical neuron pool (figure 4). Each layer 4 neuron model includes AMPAR- and NMDAR-containing synapses from the layer 6 neuron pool and AMPAR-containing synapses from the TC neuron pool, and projects AMPAR-mediated afferents to the intrinsically active cortical neuron. Layer 6 neuron model includes AMPAR- and NMDAR-containing synapses from layer 4 cells and the intrinsically active cortical neuron, and projects AMPAR- and NMDAR-containing synapses to the intrinsically active cortical neuron. Finally, the cortical intrinsically active neuron contains a recurrent NMDAR-mediated synapse.

(b) Synaptic models

The AMPAR-, NMDAR- and GABA_A_R-mediated components of our model synapses have been previously published [64,65]. The mGluR-mediated component was modelled as a first-order linear system, assuming that glutamate acts on the leakage channels by decreasing their conductance. Thus, the leakage conductance (*g*_L_) becomes dependent on the glutamate concentration. Hence

A 1

where *g*_L0_ is the control value of the leakage conductance, and *w*(*t*) is interpreted as the concentration of glutamate at the postsynaptic mGluRs. It is obtained as the solution of a first-order kinetic differential equation:

Here, *a*(*t*)=*a*_0_>0, only for a short period of time, and is 0 otherwise; *b* is the decay rate constant of *w*(*t*). Since, according to experimental findings [66] the concentration of glutamate decays only slowly at the receptors, *b* is small, of the order of 0.001 ms^−1^. In order to avoid negative conductances, we modified equation (A1) to

A 2

setting a lower bound on *g*_L_. The value of  was chosen such as to be equal to the value of *g*_L_ measured (estimated) *in vitro* in the presence of an mGlu1R agonist. Typically,  was about 1.5 nS.

The parameter values of the synaptic models were as follows.

(i) Linear synaptic models: AMPA and NMDA

These models comprise a single, linear differential equation of the form of equation (A2) [65]. For NMDA, we have: *a*_0_=0.72 ms^−1^, *b*=0.066 ms^−1^ and the extracellular concentration of Mg^2+^ was 0.1 mM. For AMPA, *a*_0_=1.1 ms^−1^ and *b*=0.19 ms^−1^ were used. In both cases, *E*_syn_=0 mV.

(ii) Nonlinear model of the GABA_A_ synapse with presynaptic inhibition

The numerical values of the parameters in the model equations are [64] *a*_low_=0.02 ms^−1^, , and *t*_ret_=5 ms; *b*_0_=5.0 ms^−1^, *c*_0_=6.0 ms^−1^, *d*_0_=1/77 ms^−1^, and *E*_syn_=−70 mV. Furthermore, in the equations of the presynaptic inhibition, we have: *a*_0_=3.0 ms^−1^, *b*=0.005 ms^−1^; *c*=0.4 ms^−1^, *d*_0_=0.004 ms^−1^ and *c*_eff_=0.92 and *K*_*d*_=0.33 mM^4^.

(iii) Model of the mGluR synapse

For the mGlu synapse, *a*_0_=0.11 ms^−1^ and *b*=9.21×10^−4^ ms^−1^ were used.

(c) Model implementation

The model was implemented in language C, and the CVODE package was used for the numerical integration of the differential equation system [67]. Simulations were carried out on a Linux cluster of 11 computers with logically dual central processing units using the Mandriva IGGI cluster software [68] and the MPI message passing software package [69].
